# Detonation Spraying of Hydroxyapatite on a Titanium Alloy Implant

**DOI:** 10.3390/ma14174852

**Published:** 2021-08-26

**Authors:** Natalia V. Bulina, Denis K. Rybin, Svetlana V. Makarova, Dina V. Dudina, Igor S. Batraev, Alexey V. Utkin, Igor Yu. Prosanov, Mikhail V. Khvostov, Vladimir Yu. Ulianitsky

**Affiliations:** 1 Institute of Solid State Chemistry and Mechanochemistry, Siberian Branch of the Russian Academy of Sciences, Kutateladze str. 18, 630128 Novosibirsk, Russia; bulina@solid.nsc.ru (N.V.B.); makarova@solid.nsc.ru (S.V.M.); utkinalex@hotmail.com (A.V.U.); prosanov@mail.ru (I.Y.P.); mihail.hvostov@gmail.com (M.V.K.); 2 Lavrentyev Institute of Hydrodynamics, Siberian Branch of the Russian Academy of Sciences, Lavrentyev Ave. 15, 630090 Novosibirsk, Russia; rybindenis1990@gmail.com (D.K.R.); ibatraev@gmail.com (I.S.B.); ulianv@mail.ru (V.Y.U.); 3 Vorozhtsov Novosibirsk Institute of Organic Chemistry, Siberian Branch of the Russian Academy of Sciences, Lavrentyev Ave. 9, 630090 Novosibirsk, Russia; 4 Siberian Branch of the Russian Academy of Sciences, Lavrentyev Ave. 17, 630090 Novosibirsk, Russia

**Keywords:** hydroxyapatite, coating, titanium, implant, detonation spraying

## Abstract

Hydroxyapatite (HA), the major mineral component of tooth enamel and natural bones, is a good candidate for bone tissue engineering. Synthetic HA is used for making coatings on metallic implants intended for medical applications. A HA coating renders the implant biocompatible and osteoinductive. In addition, it improves fixation and the overall performance of the implanted object. In the present work, HA coatings were deposited on a medical titanium alloy implant with mesh geometry and a developed surface by detonation spraying. The feedstock powder was HA obtained by the dry mechanochemical method. Single-phase HA coatings were obtained. The coatings were formed not only on the surfaces normal to the particle flow direction, but also on the sides of the mesh elements. Despite partial melting of the powder, no decomposition of HA occurred. This work demonstrates the prospects of detonation spraying for the production of HA coatings on metallic implants with complex geometries.

## 1. Introduction

Orthopedic and dental implants improve the quality of life of millions of patients every year. In recent years, the success rate of orthopedic prostheses has significantly increased owing to clinical staff training, new prosthesis designs, control of sterility, and protocols for antibiotic prophylaxis. At the same time, thousands of prostheses and dental implants must be revised every year. Failure is usually caused by insufficient bone formation around the implant immediately after implantation [1,2], especially in osteoporotic patients [3,4], or infection [5].

Synthetic materials for bone implants should possess certain surface and bulk characteristics in order to meet the biocompatibility and mechanical property requirements for a given application. Bone implants for load-bearing applications are generally made of metals or alloys (titanium, Ti-6Al-4V alloys, stainless steel). Dental implants, stems of hip and knee prostheses, plates, screws, and other fixation devices are made of metals or alloys [6].

Additive manufacturing technologies are promising for bone tissue engineering thanks to their ability to directly “print” porous implants with a pre-designed shape, controlled chemistry, and interconnected porosity [7]. Porous implants offer a significant weight reduction in comparison with dense counterparts and provide the necessary pathways for cell growth and new bone formation. However, metals alone cannot guarantee good bonding with the bone and fixation of the implant, as metallic surfaces do not form mechanically stable bonds with bone tissue. Furthermore, metallic materials release toxic metal ions and produce wear debris upon prolonged friction, causing acute or chronic responses after implantation [8].

Surface modification of metallic implants is an effective strategy to accelerate bone healing during the early implantation period. Coatings of various types have been developed to enhance the biocompatibility and osteoconductivity of implants [9,10,11]. Coating metallic implants with a layer of hydroxyapatite (HA) is one viable solution owing to the excellent biocompatibility, bioactivity, and osteoconductive behavior of HA [9]. HA is a natural biological nanomaterial and the major component of mammalian hard tissues, in which it exists either in the form of rods 20–40 nm in diameter, as in enamel, or in the form of ~5 nm-thick and 20 nm-long platelets, as in dentin and bone. The ability to accommodate ionic substitutions adds new properties to HA. These properties are determined by the nature of the substituent [12,13,14]. Therefore, coating a metal implant with HA containing substituent ions extends the list of properties useful for accelerating the healing process. For example, the presence of Ag^+^, Cu^2+^ or Zn^2+^ cations provides antibacterial properties, while CO_3_^2−^, SiO_4_^4−^ and Mg^2+^ stimulate new bone growth [13,15]. On the implanted part, a HA coating can be replaced by autologous bone, as, similar to natural bone, HA participates in the bone remodeling response.

A review on the calcium orthophosphate deposition techniques [9] shows that there are about fifty different approaches to performing the deposition, among which there are different thermal spray methods. Each deposition technique has its own advantages and disadvantages [9]. Since none of the methods are able to ensure perfect coating, the deposited layers always have some imperfections, such as cracks, pores, or second phases, and suffer from residual stresses and/or poor adhesion. All these imperfections reduce the durability of the coatings, leading to partial or complete destruction in body fluids over time.

Until now, very few studies have dealt with detonation spraying of HA [16,17,18,19,20]. This method features high productivity and allows coatings of variable thickness and high adhesion to the substrate to be obtained. An amorphous phase and the products of decomposition of HA can be present in the coatings, along with crystalline HA [16,17,18,19,20]. During the detonation spraying process, some particles are heated to temperatures exceeding the melting point of HA. Upon deposition, the melt is rapidly cooled, which can lead to the formation of an amorphous phase [17]. Low crystallinity and high residual stresses cause fast dissolution of the deposited layers [18,19]. Detonation spraying allows coating not only flat surfaces, but also surfaces with complex geometries [21].

**Figure 1:**
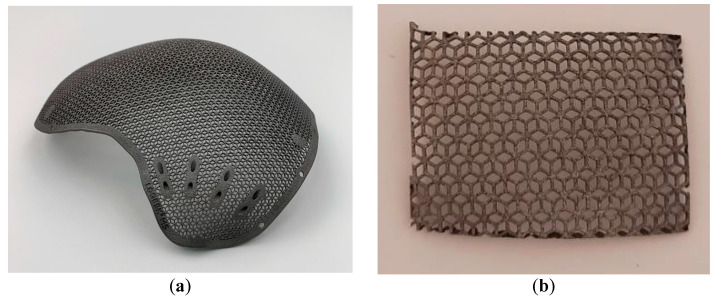
An implant for restoring the integrity and anatomical shape of the skull with a structure analogous to the mesh structure of the samples used in the present work (**a**). General view of a titanium plate of mesh structure, onto which the HA coating was deposited (**b**).

The present work reports the morphology and structure of HA coatings formed on the surface of a medical implant with a complex mesh structure (Figure 1a) by detonation spraying.

## 2. Materials and Methods

The HA powder was synthesized by the dry mechanochemical method via mechanical treatment of a reaction mixture composed of CaHPO_4_ and calcined CaO powders. The reagents were mixed in ratios according to the following equation:

6CaHPO_4_ + 4CaO → Ca_10_(PO_4_)_6_(OH)_2_ + 2H_2_O → Ca_10_(PO_4_)_6_(OH)_2_ · 2H_2_O (1)

Mechanical treatment of the mixture was carried out in a planetary ball mill (AGO-2) equipped with water-cooled steel vials. The reaction mixture was treated by steel balls (total weight 200 g) at a rotational speed of 1800 rpm. The duration of the synthesis was 30 min. A portion of the as-synthesized powder was calcined in a high-temperature PVK-1.6-5 electrical furnace at 1000 °C for 2 h (heating rate up to the maximum temperature was 5 °C min^−1^). Both the as-synthesized powder (nano-HA) and calcined powder (calc-HA) were sieved to separate the 64–100 μm fraction.

A 3D-printed mesh plate (Figure 1b) was supplied by LLC “LOGEEKS MEDICAL SYSTEMS”. A titanium alloy powder (Ti-6Al-4V) was used as a feedstock material. The mesh plate was formed by direct metal laser sintering [22] using an EOS M290 metal 3D printer (EOS GmbH).

The printed objects satisfy international standards [23,24,25] and manufacturer specifications. The substrate plate used in this work is analogous to real implants, having individually designed anatomical shapes (Figure 1b) in terms of structure, architecture, and finishing.

HA coatings were deposited on the titanium alloy substrates using a computer-controlled detonation spraying facility (CCDS2000) [26,27]. The explosive mixture used for spraying had a stoichiometry of C_2_H_2_ + 3.6 O_2_. Nitrogen was used as a carrier gas. The powders of nano-HA and calc-HA were deposited on mesh plates with dimensions of 10 mm × 10 mm. The coatings were formed via 200 shots of the detonation gun at a speed of 2 shots per second.

Thermal analysis of the HA powders was carried out on a STA 449 F1 Jupiter apparatus (NETZSCH, Selb, Germany) equipped with a QMS 403 C Aeolos mass spectrometer (NETZSCH, Selb, Germany).

X-ray diffraction (XRD) patterns of the powders and coatings were recorded on a D8 Advance powder diffractometer (Bruker, Karlsruhe, Germany) with Bragg–Brentano geometry using Cu Kα radiation. XRD phase analysis of the powders and coatings was carried out using the PDF-4 database (ICDD, 2011). The unit cell parameters and crystallite size were refined by the Rietveld method using Topas 4.2 software (Bruker, Karlsruhe, Germany).

Fourier transform infrared (FTIR) spectra of the powders and coatings were recorded on an Infralum FT-801 spectrometer (Simex, Novosibirsk, Russia). The specimens were prepared by the KBr pellet method.

The substrate/coatings samples were mounted into epoxy resin and polished with polycrystalline diamond suspensions to prepare cross-sections for microstructure observations and microhardness measurements. The surface morphology and microstructure of the samples were examined using scanning electron microscopy on a TM-1000 Tabletop microscope (Hitachi, Tokyo, Japan).

The microhardness of the HA coatings was measured on the cross-section (front surface deposition) using DuraScan-50, a Vickers hardness testing device (EMCO-TEST, Kuchl, Austria), at a load of 0.098 N and a dwell time of 10 s. At least 10 indents were made for each sample. The average microhardness values are reported along with standard deviations.

## 3. Results and Discussion

### 3.1. Characterization of HA Feedstock Powders

**Figure 2:**
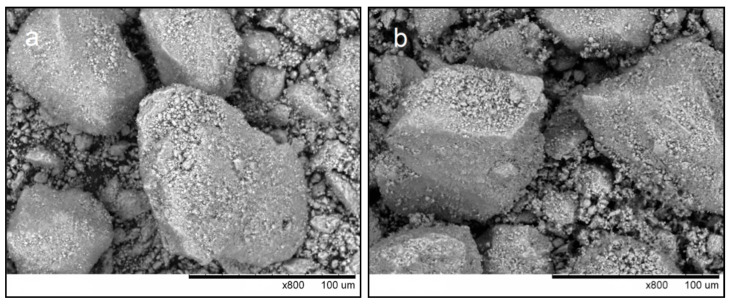
SEM images of the feedstock powders: (**a**) nano-HA; (**b**) calc-HA.

Figure 2 shows the morphology of nano-HA and calc-HA powders. It is seen that the powders possess similar morphological features, indicating that, during annealing of the mechanochemically synthesized HA, there was little particle shape change, if any.

**Figure 3:**
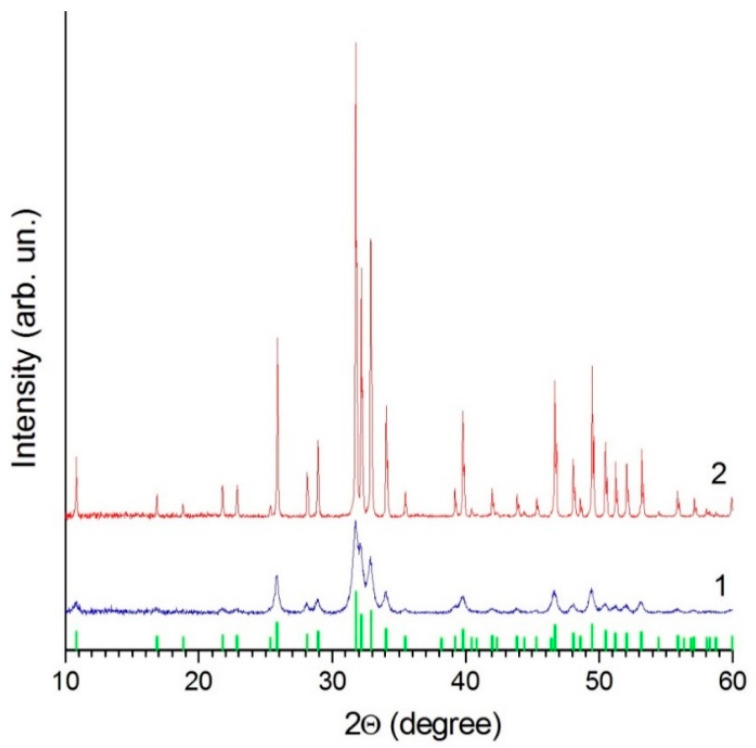
XRD patterns of nano-HA (1) and calc-HA (2) powders. The bar chart corresponds to PDF file 40-11-9308 (ICDD PDF-4) of crystalline HA.

The powders do not contain phases other than HA (Figure 3). All observed reflections correspond to the HA crystal structure (PDF file 40-11-9308, ICDD PDF-4). The lattice parameters of HA in nano-HA are larger than in calc-HA (Table 1). This difference is due to the presence of lattice water molecules in nano-HA forming as a product in reaction (1). The crystallite size of nano-HA was calculated to be 24 nm. The size of the crystallites increases one order of magnitude upon annealing.

**Figure 4:**
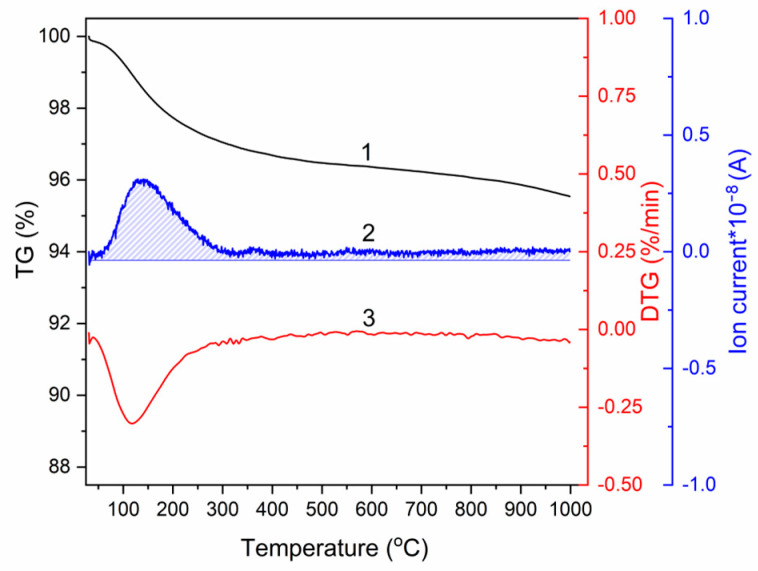
Thermal analysis of nano-HA: 1—thermogravimetry (TG); 2—mass spectrum indicating water release; 3—differential thermogravimetric curve (DTG).

Results of the thermogravimetric analysis show that absorbed water and lattice water are released in the 70–300 °C temperature range, accounting for a weight loss of 2.7% (Figure 4). Water release from the as-synthesized product upon heating was also confirmed by mass spectrometry. Therefore, nano-HA and calc-HA powders have the same phase composition but different sizes of crystallites and different lattice parameters. The lattice and absorbed water are present in the as-synthesized powder.

### 3.2. Characterization of Detonation Coatings

**Figure 5:**
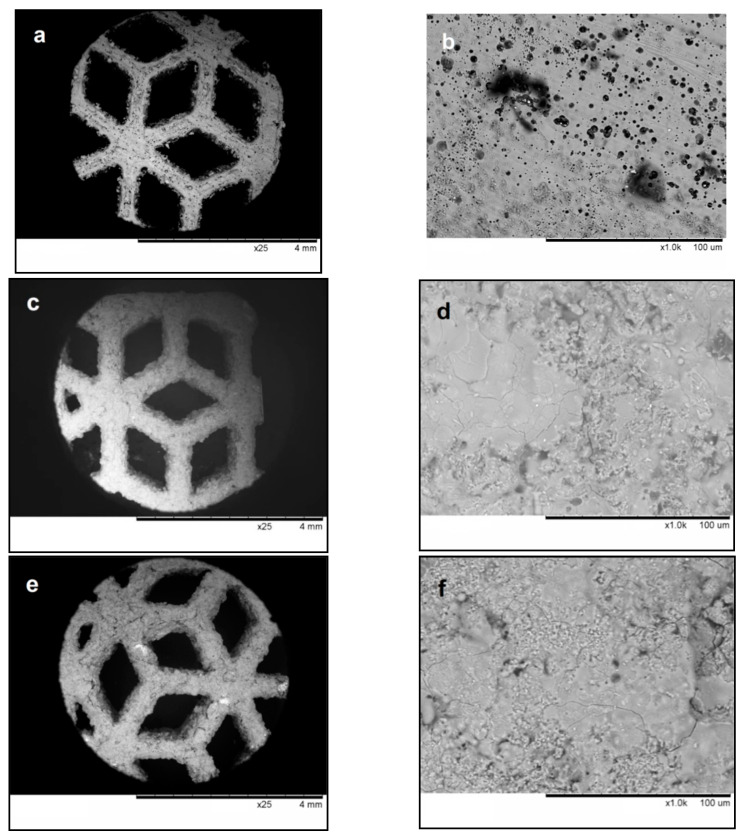
SEM images of the surface of the as-printed mesh plate (**a**,**b**) and plates coated by nano-HA (**c**,**d**) and calc-HA (**e**,**f**). General view (**a**,**c**,**e**) and surface morphology (**b**,**d**,**f**).

The general view and surface morphology of the titanium alloy mesh substrate are shown in Figure 5a,b. The elements of the mesh have rough edges. The surface of the alloy is not perfectly flat; defects of hollow shape (about 10 μm in size) are visible.

**Figure 6:**
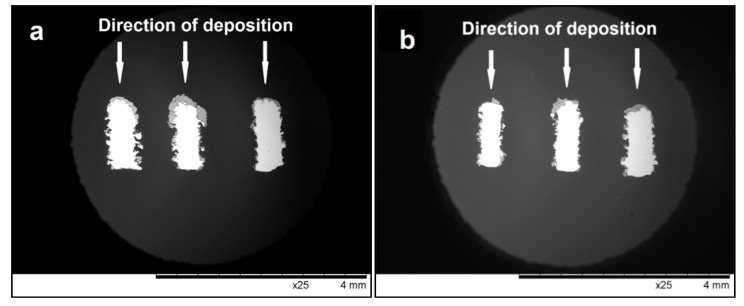

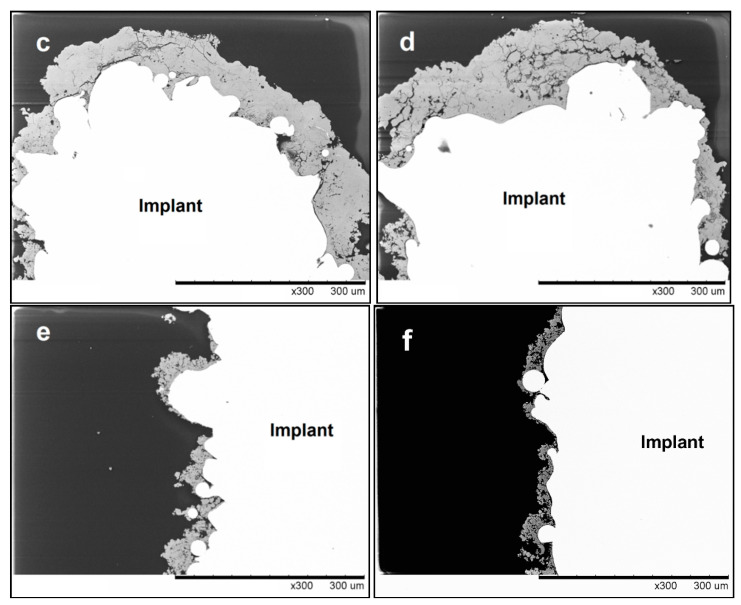
SEM images of cross-sections of the mesh with coatings obtained from nano-HA (**a**,**c**,**e**) and calc-HA (**b**,**d**,**f**). General view (**a**,**b**), coatings on front (**c**,**d**) and side (**e**,**f**) surfaces.

During detonation spraying, the surface of the mesh is fully coated (Figure 5c,e). The surfaces of the coatings formed from both powders show areas of resolidified melt (Figure 5d,f). This surface morphology has been observed by other authors [18] and is explained by melting of the particles during spraying. The relative density of the coatings formed by partially molten particles is usually higher than that of coatings formed by deposition in the solid state. The possibility of coating deposition under conditions of partial melting of the material is an advantage of the detonation spraying technique. As discussed above, the HA feedstock powders used in the present work differed by the size of the crystallites and the presence of lattice/absorbed water. As the morphology of the coatings obtained from nano-HA was similar to that of the coating obtained from calc-HA, it appears that those characteristics did not significantly influence the surface morphology of the detonation coatings. The cross-sectional view of the mesh elements (Figure 6) shows that its surface is very complex (uneven and tortuous).

The HA layer formed by detonation spraying almost fully coats the front surface of the mesh and has a varying thickness, which does not exceed 150 μm. Within the coating volume, there are pores, cracks, and voids, which are beneficial for the processes of cell growth and new bone formation. At the same time, there are no pores at the coating/substrate interface, which is a promising feature for good integrity of the system as a whole. Notably, the side surface of the mesh elements (surfaces parallel to the particle flow direction) was also coated in the spraying experiments; however, the coating was thinner in those areas (Figure 6e,f). The coating thickness on the sides of the mesh elements was below 30 μm. The formation of the coating on the side surface is an advantage of detonation spraying, making it a suitable method for depositing coatings on the surfaces of real implants, which are usually tortuous (Figure 1b).

In Figure 6, one can see that the coating has a non-constant thickness. The deposition efficiency is higher for surfaces normal to the direction of particle flight than for surfaces parallel to this direction (particles attach to the surface normal to the particle flight direction with a higher probability). In the process of bioresorption of coatings with variable thickness, thinner areas will dissolve faster and, in those areas, the process of osseointegration of the metal implant will begin. Areas with a thicker coating will be a long-term source of ions necessary for the formation of the mineral component of new bone tissue.

The values of microhardness of the coatings formed on the front surface of the mesh from nano-HA and calc-HA powders are close to each other; the hardness of the coating formed from nano-HA is 6.3 ± 0.6 GPa, while that of the coating formed from calc-HA is 7.1 ± 0.5 GPa.

**Figure 7:**
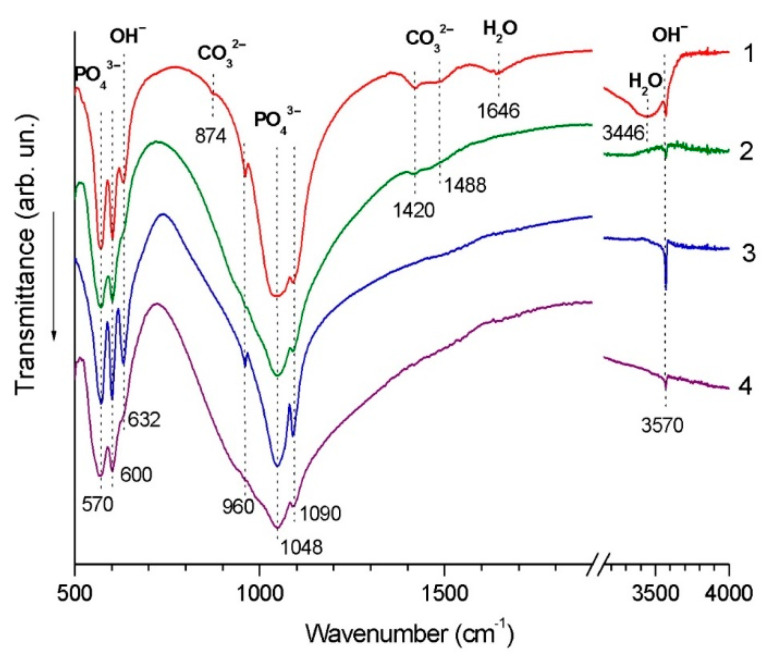
FTIR spectra of nano-HA powder (1), detonation coating obtained from nano-HA powder (2), calc-HA powder (3), detonation coating obtained from calc-HA powder (4).

Figure 7 shows the FTIR spectra of the deposited coatings along with the spectra of the powders. From the comparison, one can see that, despite differences in the spectra of the feedstock powders, the spectra of the coatings are identical. The absorption bands do not shift after deposition. The characteristic bands of PO_4_^3−^ ions, namely, bending vibrations of the O–P–O bond (570 cm^−1^ and 600 cm^−1^) and stretching vibrations of the P–O bond (960 cm^−1^, 1048 cm^−1^ and 1090 cm^−1^), become broader in the spectra of the coatings. Furthermore, the intensities of the stretching bands at 3570 cm^−1^ and libration bands at 632 cm^−1^ originating from OH^−^ groups are lower in the spectra of the coatings, which indicates a decrease in the number of hydroxyl groups. It should also be noted that in the spectrum of the coating obtained from nano-HA powder, there are no absorption bands of the sorbed water (1646 cm^−1^ and 3446 cm^−1^) and practically no absorption bands of the carbonate ion (1420 cm^−1^ and 1488 cm^−1^), which were present in the spectrum of the feedstock powder. These species evaporated during detonation spraying.

**Figure 8:**
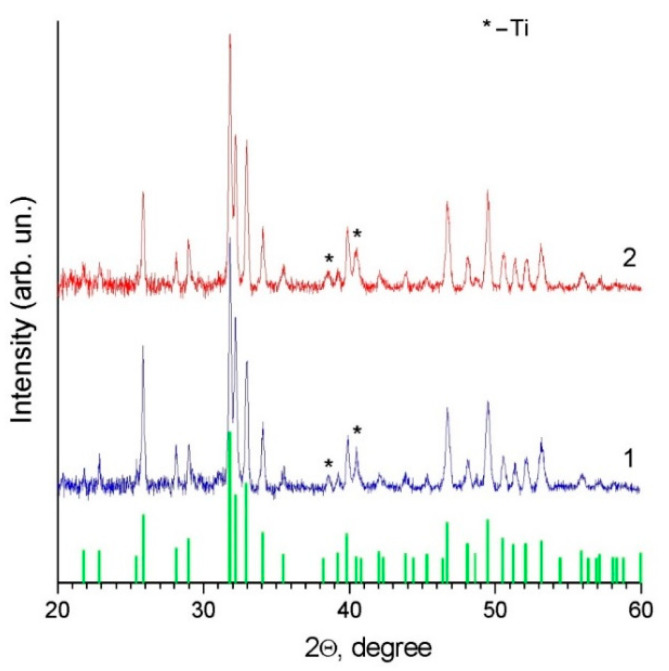
XRD patterns of the titanium alloy mesh with detonation coatings obtained from nano-HA (1) and calc-HA (2). Bar chart corresponds to PDF file 40-11-9308 (ICDD PDF-4) of crystalline HA.

XRD phase analysis of the coating shows that, in both cases, the coating is single-phase. The XRD patterns of the coated samples (Figure 8) demonstrate only reflections of HA and metallic substrate (titanium).

The crystallite sizes and lattice parameters of coatings obtained from nano-HA and calc-HA are close to each other, despite significant differences in the values of these parameters in the powder state (Table 1). Consequently, heating of the powder during spraying eliminates the differences between the two powders. The formation of detonation coatings with a crystallite size finer than that of the powder implies the operation of a structure refinement mechanism during spraying. Upon heating in a flow of hot gases, HA particles partially melt, as confirmed by morphological investigations of the coating surface of samples produced from both nano-HA and calc-HA. So, the formation of detonation coatings with a fine crystallite size can be due to melting and rapid cooling of the splats. In addition, heating of the particles during detonation spraying leads to partial loss of the OH groups, enabling the transition of HA to an oxyhydroxy form, namely, to oxyhydroxyapatite, in accordance with the following reaction:

Ca_10_(PO_4_)_6_(OH)_2_ → Ca_10_(PO_4_)_6_(OH)_2−2x_O_x_□_x_ + xH_2_O, (2)

where □ is a vacancy of the OH group. The loss of OH groups, which was confirmed by FTIR spectroscopy, appears to contribute to the microstructure refinement of the deposited material. This transformation leads to changes in the lattice parameters of HA; the value of *a* decreases, while the value of *c* increases. The growth of HA crystallites is hindered by reaction (2), as partial elimination of OH groups increases the concentration of defects in the crystals. The molten part of the material crystallizes using colder particles as crystallization centers, forming an OH-depleted structure. Considering the fact that oxyapatite has a much higher solubility than HA, which is practically insoluble [9], it can be assumed that oxyhydroxyapatite is more soluble than HA and is a promising candidate for bioresorbable coatings.

In refs. [16,18,20], an amorphous phase was detected in the detonation-sprayed HA coatings. In the present study, a halo corresponding to an amorphous phase was not detected in the XRD patterns. However, the presence of certain amounts of an amorphous phase cannot be excluded, as the detection limit of the XRD phase analysis method is ~5%. It should be noted that the formation of metastable phases in detonation coatings is influenced by the cooling rates of the splats, which, in turn, depend on particle velocities [28]. Faster-moving particles produce thinner splats upon impact with the substrate or the previously deposited layers of the coating. The differences in the phase composition of coatings obtained by different authors [16,17,18,19,20] can be due to different temperature–velocity combinations involved in the spraying process. The similarity of the characteristics of coatings obtained by nano-HA and calc-HA indicates that neither the crystallite size of the feedstock powder nor absorbed/lattice water influences the structural features of the detonation coating.

Upon heating of HA in a furnace with a heating rate of 5 °C min^−1^ or lower up to 1300 °C, partial decomposition of HA occurs, such that Ca_3_(PO_4_)_2_ and Ca_4_O(PO_4_)_2_ phases form [29]. The decomposition reaction does not allow the realization of congruent melting of the compound. However, congruent melting is possible upon treatment of HA with laser irradiation [30], owing to fast heating of the material occurring within milliseconds. Therefore, the HA structure does not have enough time to experience decomposition. In a similar manner, in detonation spraying, the particles interact with the hot gases within 2–5 ms [27], which makes it possible for HA to melt congruently during the process.

## 4. Conclusions

This work has shown that, using detonation spraying, it is possible to deposit HA on metallic substrates with complex geometries and surface morphologies. HA coatings with a porous structure have been obtained on 3D-printed substrates of mesh geometry made of Ti-6Al-4V alloy by direct metal laser sintering. The coatings were characterized by XRD phase analysis and were found to be single-phase oxyhydroxyapatite Ca_10_(PO_4_)_6_(OH)_2−2x_O_x_□_x_ with an average size of crystallites of ~65 nm. The surface of the coatings revealed both resolidified regions and particles that did not experience melting. On the substrate, the melt crystallized upon cooling, the solid particles present acting as centers of crystallization. No decomposition products of HA or amorphous phases were detected in the coatings by XRD phase analysis or FTIR spectroscopy. As the crystallite size of the HA feedstock powder and absorbed/lattice water in the powder do not influence the structural features of the detonation coating, the mechanochemically synthesized HA powder can be used as a feedstock material in the detonation spraying process without an additional preliminary heat treatment.

## Figures and Tables

**Table 1:** Structural parameters of the HA phase in powders and coatings.

Powder/Coating	*a* (Å)	*c* (Å)	Crystallite Size (nm)
nano-HA powder	9.434 ± 0.001	6.8912 ± 0.0008	24.2 ± 0.04
calc-HA powder	9.4243 ± 0.0002	6.8816 ± 0.0002	234 ± 6
coating from nano-HA powder	9.410 ± 0.002	6.890 ± 0.002	64 ± 3
coating from calc-HA powder	9.411 ± 0.003	6.891 ± 0.002	66 ± 4

## Data Availability

The raw/processed data required to reproduce these results are included in the Materials and Methods section.
